# Methyl 4,6-dichloro­pyridine-3-carboxyl­ate

**DOI:** 10.1107/S1600536808011914

**Published:** 2008-05-03

**Authors:** Yi Ma, Jun Liu

**Affiliations:** a School of Chemical Engineering, Shandong Institute of Light Industry, Jinan 250353, People’s Republic of China; b Jinan Sijian (Group) Co. Ltd, Jinan 250031, People’s Republic of China

## Abstract

The title compound, C_7_H_5_Cl_2_NO_2_, crystallizes with two independent mol­ecules in the asymmetric unit. The bond lengths and angles in both mol­ecules are within normal ranges. In the crystal structure, weak inter­molecular C—H⋯O hydrogen bonds link the mol­ecules into layers parallel to the [010] plane.

## Related literature

For details of the biological activity of the title compound, see: Wallace *et al.* (2006 ▶); Bondinell *et al.* (2002 ▶). For a related structure, see: McArdle *et al.* (1982 ▶).
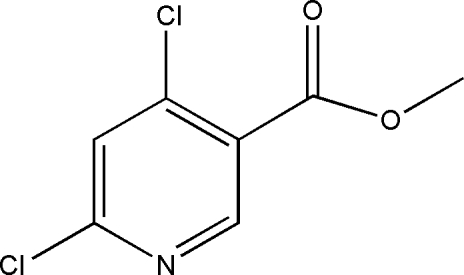


## Experimental

### 

#### Crystal data

C_7_H_5_Cl_2_NO_2_
*M* *_r_* = 206.02
Monoclinic, 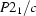

*a* = 8.033 (4) Å
*b* = 18.974 (9) Å
*c* = 11.240 (6) Å
β = 95.224 (8)°
*V* = 1705.9 (15) Å^3^
*Z* = 8
Mo *K*α radiation
μ = 0.71 mm^−1^
*T* = 298 (2) K
0.45 × 0.19 × 0.06 mm

#### Data collection

Bruker SMART CCD area-detector diffractometer
Absorption correction: multi-scan (*SADABS*; Sheldrick, 2004 ▶) *T*_min_ = 0.739, *T*_max_ = 0.958
8532 measured reflections
3012 independent reflections
2289 reflections with *I* > 2σ(*I*)
*R*_int_ = 0.035

#### Refinement

*R*[*F* ^2^ > 2σ(*F* ^2^)] = 0.052
*wR*(*F* ^2^) = 0.135
*S* = 1.08
3012 reflections
217 parameters
H-atom parameters constrained
Δρ_max_ = 0.25 e Å^−3^
Δρ_min_ = −0.18 e Å^−3^

### 

Data collection: *SMART* (Bruker, 2001 ▶); cell refinement: *SAINT* (Bruker, 2001 ▶); data reduction: *SAINT*; program(s) used to solve structure: *SHELXTL* (Sheldrick, 2008 ▶); program(s) used to refine structure: *SHELXTL*; molecular graphics: *SHELXTL*; software used to prepare material for publication: *SHELXTL* and local programs.

## Supplementary Material

Crystal structure: contains datablocks I, global. DOI: 10.1107/S1600536808011914/hg2396sup1.cif

Structure factors: contains datablocks I. DOI: 10.1107/S1600536808011914/hg2396Isup2.hkl

Additional supplementary materials:  crystallographic information; 3D view; checkCIF report

## Figures and Tables

**Table 1:** Hydrogen-bond geometry (Å, °)

*D*—H⋯*A*	*D*—H	H⋯*A*	*D*⋯*A*	*D*—H⋯*A*
C6—H6*A*⋯O3^i^	0.93	2.41	3.309 (4)	162
C11—H11*A*⋯O2^ii^	0.93	2.60	3.513 (4)	168

Symmetry codes: (i) 
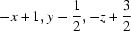
; (ii) 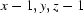.

**Fig. 1.**
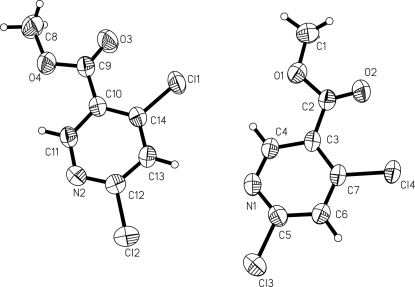
View of the title compound (I), with displacement ellipsoids drawn at the 40% probability level.

## supplementary crystallographic information

### Comment

Methyl 4,6-dichloropyridine-3-carboxylate is a useful intermediate for the synthesis of different kinase inhibitors (Wallace *et al.*, 2006; Bondinell *et al.*, 2002). In this paper, we report the crystal structure of the title compound (I).

Compound (I) crystallizes with two independent molecules in the asymmetric unit (Fig. 1), all bond lengths and angles are normal and in a good agreement with those reported previously (McArdle *et al.*, 1982). The dihedral angles between the planes of the methoxycarbonyl group (C1/C2/O1/O2; C8/C9/O3/O4) and pyridine rings in the two independent molecules are 10.9 (2) and 8.1 (4)°. In the crystal structure, weak intermolecular C—H···O hydrogen bonds link the molecules into layers parallel to the *b* axis.

### Experimental

Methyl 4, 6-dichloropyridine-3-carboxylate was synthesized from Methyl 4, 6-dihydroxypyridine-3-carboxylate *via* chlorination with POCl_3_. The desired compound was obtained as a low melting yellow solid in 89% yield. Crystals suitable for X-ray diffraction analysis were obtained by slow evaporation of a solution in a hexane/dichloromethane mixture (1: 4 *v*/*v*) at room temperature over a period of one week.

### Refinement

All H atoms were placed in calculated positions, with C—H = 0.93 or 0.96 Å, and included in the final cycles of refinement using a riding model, with *U*_iso_(H) = 1.2 times *U*_eq_(C).

### Crystal data

**Table:**

C_7_H_5_Cl_2_NO_2_	*F*_000_ = 832
*M**_r_* = 206.02	*D*_x_ = 1.604 Mg m^−^^3^
Monoclinic, *P*2_1_/*c*	Mo *K*α radiation λ = 0.71073 Å
Hall symbol: -P 2ybc	Cell parameters from 1274 reflections
*a* = 8.033 (4) Å	θ = 2.8–27.9º
*b* = 18.974 (9) Å	µ = 0.72 mm^−^^1^
*c* = 11.240 (6) Å	*T* = 298 (2) K
β = 95.224 (8)º	Block, colorless
*V* = 1705.9 (15) Å^3^	0.45 × 0.19 × 0.06 mm
*Z* = 8	

### Data collection

**Table:**

Bruker SMART CCD area-detector diffractometer	3012 independent reflections
Radiation source: fine-focus sealed tube	2289 reflections with *I* > 2σ(*I*)
Monochromator: graphite	*R*_int_ = 0.035
*T* = 298(2) K	θ_max_ = 25.0º
φ and ω scans	θ_min_ = 2.1º
Absorption correction: multi-scan(SADABS; Sheldrick, 2004)	*h* = −9→9
*T*_min_ = 0.739, *T*_max_ = 0.958	*k* = −16→22
8532 measured reflections	*l* = −12→13

### Refinement

**Table:**

Refinement on *F*^2^	Secondary atom site location: difference Fourier map
Least-squares matrix: full	Hydrogen site location: inferred from neighbouring sites
*R*[*F*^2^ > 2σ(*F*^2^)] = 0.052	H-atom parameters constrained
*wR*(*F*^2^) = 0.135	*w* = 1/[σ^2^(*F*_o_^2^) + (0.0645*P*)^2^ + 0.3441*P*] where *P* = (*F*_o_^2^ + 2*F*_c_^2^)/3
*S* = 1.08	(Δ/σ)_max_ = 0.001
3012 reflections	Δρ_max_ = 0.25 e Å^−^^3^
217 parameters	Δρ_min_ = −0.18 e Å^−^^3^
Primary atom site location: structure-invariant direct methods	Extinction correction: none

### Special details

**Table:**

Geometry. All e.s.d.'s (except the e.s.d. in the dihedral angle between two l.s. planes) are estimated using the full covariance matrix. The cell e.s.d.'s are taken into account individually in the estimation of e.s.d.'s in distances, angles and torsion angles; correlations between e.s.d.'s in cell parameters are only used when they are defined by crystal symmetry. An approximate (isotropic) treatment of cell e.s.d.'s is used for estimating e.s.d.'s involving l.s. planes.
Refinement. Refinement of *F*^2^ against ALL reflections. The weighted *R*-factor *wR* and goodness of fit *S* are based on *F*^2^, conventional *R*-factors *R* are based on *F*, with *F* set to zero for negative *F*^2^. The threshold expression of *F*^2^ > σ(*F*^2^) is used only for calculating *R*-factors(gt) *etc*. and is not relevant to the choice of reflections for refinement. *R*-factors based on *F*^2^ are statistically about twice as large as those based on *F*, and *R*- factors based on ALL data will be even larger.

### Fractional atomic coordinates and isotropic or equivalent isotropic displacement parameters (Å^2^)

**Table:**

	*x*	*y*	*z*	*U*_iso_*/*U*_eq_	
Cl1	0.31475 (11)	0.33695 (5)	0.64891 (7)	0.0715 (3)	
Cl4	0.81189 (12)	0.14450 (4)	1.15267 (7)	0.0730 (3)	
Cl3	0.49419 (14)	0.01243 (5)	0.78099 (8)	0.0883 (4)	
Cl2	0.16454 (16)	0.07877 (5)	0.49234 (11)	0.1033 (4)	
O4	0.0012 (3)	0.39718 (12)	0.3185 (2)	0.0810 (7)	
O1	0.6147 (3)	0.33347 (11)	0.9518 (2)	0.0761 (7)	
C3	0.6515 (3)	0.21244 (15)	0.9614 (2)	0.0494 (7)	
C4	0.5571 (4)	0.20765 (17)	0.8514 (3)	0.0629 (8)	
H4A	0.5253	0.2497	0.8133	0.075*	
O2	0.7987 (3)	0.29383 (12)	1.0946 (2)	0.0859 (8)	
C6	0.6496 (4)	0.08658 (16)	0.9608 (3)	0.0586 (8)	
H6A	0.6799	0.0434	0.9955	0.070*	
N1	0.5088 (4)	0.14837 (15)	0.7965 (2)	0.0676 (7)	
C10	0.1250 (3)	0.30967 (15)	0.4395 (2)	0.0496 (7)	
C11	0.0479 (4)	0.25996 (16)	0.3617 (3)	0.0570 (8)	
H11A	−0.0152	0.2765	0.2940	0.068*	
C13	0.2303 (4)	0.21170 (16)	0.5586 (3)	0.0585 (8)	
H13A	0.2924	0.1930	0.6251	0.070*	
O3	0.1841 (4)	0.43167 (13)	0.4631 (2)	0.1010 (10)	
C5	0.5558 (4)	0.09031 (17)	0.8522 (3)	0.0603 (8)	
C7	0.6965 (4)	0.14869 (15)	1.0159 (2)	0.0504 (7)	
C9	0.1096 (4)	0.38562 (17)	0.4109 (3)	0.0574 (8)	
C1	0.6574 (6)	0.40446 (18)	0.9884 (4)	0.0940 (13)	
H1B	0.5889	0.4371	0.9405	0.141*	
H1C	0.7730	0.4131	0.9781	0.141*	
H1D	0.6388	0.4105	1.0710	0.141*	
N2	0.0578 (3)	0.19055 (14)	0.3773 (2)	0.0640 (7)	
C14	0.2165 (4)	0.28322 (15)	0.5410 (2)	0.0505 (7)	
C2	0.6989 (4)	0.28237 (16)	1.0116 (3)	0.0552 (7)	
C8	−0.0218 (5)	0.46966 (19)	0.2809 (3)	0.0893 (12)	
H8A	−0.1038	0.4719	0.2133	0.134*	
H8B	0.0823	0.4883	0.2591	0.134*	
H8C	−0.0592	0.4969	0.3453	0.134*	
C12	0.1485 (4)	0.16916 (16)	0.4737 (3)	0.0612 (8)	

### Atomic displacement parameters (Å^2^)

**Table:**

	*U*^11^	*U*^22^	*U*^33^	*U*^12^	*U*^13^	*U*^23^
Cl1	0.0777 (6)	0.0746 (6)	0.0578 (5)	−0.0105 (4)	−0.0170 (4)	−0.0072 (4)
Cl4	0.0903 (7)	0.0682 (5)	0.0546 (5)	0.0025 (4)	−0.0261 (4)	0.0098 (4)
Cl3	0.1127 (9)	0.0703 (6)	0.0774 (6)	−0.0146 (5)	−0.0161 (5)	−0.0120 (4)
Cl2	0.1327 (10)	0.0557 (5)	0.1145 (8)	0.0077 (6)	−0.0265 (7)	−0.0019 (5)
O4	0.0964 (19)	0.0624 (14)	0.0776 (15)	−0.0033 (13)	−0.0278 (14)	0.0115 (12)
O1	0.0920 (18)	0.0560 (13)	0.0748 (15)	0.0027 (12)	−0.0222 (12)	0.0056 (11)
C3	0.0457 (17)	0.0590 (18)	0.0425 (15)	0.0017 (13)	−0.0020 (12)	0.0062 (13)
C4	0.072 (2)	0.0584 (19)	0.0548 (18)	−0.0003 (16)	−0.0144 (15)	0.0074 (15)
O2	0.107 (2)	0.0698 (16)	0.0729 (15)	−0.0033 (14)	−0.0369 (14)	−0.0034 (12)
C6	0.064 (2)	0.0565 (18)	0.0530 (18)	0.0022 (15)	−0.0044 (15)	0.0079 (14)
N1	0.076 (2)	0.0683 (18)	0.0538 (15)	−0.0015 (14)	−0.0175 (13)	0.0034 (13)
C10	0.0448 (17)	0.0594 (18)	0.0444 (15)	−0.0022 (14)	0.0035 (12)	−0.0019 (13)
C11	0.059 (2)	0.064 (2)	0.0466 (16)	−0.0005 (15)	−0.0042 (14)	−0.0028 (14)
C13	0.0565 (19)	0.066 (2)	0.0509 (17)	0.0018 (15)	−0.0056 (14)	0.0038 (14)
O3	0.136 (3)	0.0614 (16)	0.0958 (19)	−0.0204 (15)	−0.0430 (18)	0.0030 (13)
C5	0.064 (2)	0.063 (2)	0.0528 (17)	−0.0050 (16)	−0.0029 (15)	−0.0045 (15)
C7	0.0465 (17)	0.0616 (18)	0.0418 (15)	0.0028 (14)	−0.0031 (12)	0.0062 (13)
C9	0.058 (2)	0.064 (2)	0.0501 (17)	−0.0045 (16)	−0.0014 (15)	0.0015 (15)
C1	0.133 (4)	0.053 (2)	0.090 (3)	0.003 (2)	−0.020 (2)	−0.0003 (18)
N2	0.0729 (19)	0.0568 (16)	0.0602 (16)	−0.0026 (13)	−0.0062 (13)	−0.0087 (13)
C14	0.0462 (17)	0.0586 (18)	0.0464 (16)	−0.0040 (13)	0.0034 (13)	−0.0036 (13)
C2	0.061 (2)	0.0597 (18)	0.0445 (16)	0.0022 (15)	0.0002 (14)	0.0054 (14)
C8	0.105 (3)	0.068 (2)	0.090 (3)	0.002 (2)	−0.016 (2)	0.0210 (19)
C12	0.064 (2)	0.0546 (18)	0.064 (2)	0.0040 (15)	−0.0002 (16)	−0.0012 (15)

### Geometric parameters (Å, °)

**Table:**

Cl1—C14	1.720 (3)	N1—C5	1.306 (4)
Cl4—C7	1.723 (3)	C10—C11	1.392 (4)
Cl3—C5	1.731 (3)	C10—C14	1.393 (4)
Cl2—C12	1.731 (3)	C10—C9	1.479 (4)
O4—C9	1.313 (4)	C11—N2	1.330 (4)
O4—C8	1.446 (4)	C11—H11A	0.9300
O1—C2	1.329 (4)	C13—C12	1.371 (4)
O1—C1	1.441 (4)	C13—C14	1.374 (4)
C3—C7	1.389 (4)	C13—H13A	0.9300
C3—C4	1.393 (4)	O3—C9	1.184 (4)
C3—C2	1.478 (4)	C1—H1B	0.9600
C4—N1	1.324 (4)	C1—H1C	0.9600
C4—H4A	0.9300	C1—H1D	0.9600
O2—C2	1.194 (4)	N2—C12	1.314 (4)
C6—C7	1.368 (4)	C8—H8A	0.9600
C6—C5	1.376 (4)	C8—H8B	0.9600
C6—H6A	0.9300	C8—H8C	0.9600
			
C9—O4—C8	116.6 (3)	C3—C7—Cl4	122.1 (2)
C2—O1—C1	116.2 (3)	O3—C9—O4	122.6 (3)
C7—C3—C4	115.7 (3)	O3—C9—C10	125.7 (3)
C7—C3—C2	124.4 (2)	O4—C9—C10	111.8 (3)
C4—C3—C2	119.8 (3)	O1—C1—H1B	109.5
N1—C4—C3	125.6 (3)	O1—C1—H1C	109.5
N1—C4—H4A	117.2	H1B—C1—H1C	109.5
C3—C4—H4A	117.2	O1—C1—H1D	109.5
C7—C6—C5	117.6 (3)	H1B—C1—H1D	109.5
C7—C6—H6A	121.2	H1C—C1—H1D	109.5
C5—C6—H6A	121.2	C12—N2—C11	115.9 (3)
C5—N1—C4	115.7 (3)	C13—C14—C10	120.2 (3)
C11—C10—C14	116.2 (3)	C13—C14—Cl1	117.2 (2)
C11—C10—C9	120.0 (3)	C10—C14—Cl1	122.5 (2)
C14—C10—C9	123.8 (3)	O2—C2—O1	122.5 (3)
N2—C11—C10	124.8 (3)	O2—C2—C3	126.4 (3)
N2—C11—H11A	117.6	O1—C2—C3	111.2 (3)
C10—C11—H11A	117.6	O4—C8—H8A	109.5
C12—C13—C14	117.0 (3)	O4—C8—H8B	109.5
C12—C13—H13A	121.5	H8A—C8—H8B	109.5
C14—C13—H13A	121.5	O4—C8—H8C	109.5
N1—C5—C6	125.4 (3)	H8A—C8—H8C	109.5
N1—C5—Cl3	116.1 (2)	H8B—C8—H8C	109.5
C6—C5—Cl3	118.5 (2)	N2—C12—C13	125.9 (3)
C6—C7—C3	120.0 (3)	N2—C12—Cl2	115.8 (2)
C6—C7—Cl4	117.9 (2)	C13—C12—Cl2	118.2 (2)
			
C7—C3—C4—N1	−0.2 (5)	C11—C10—C9—O4	8.7 (4)
C2—C3—C4—N1	179.1 (3)	C14—C10—C9—O4	−172.4 (3)
C3—C4—N1—C5	−0.1 (5)	C10—C11—N2—C12	−0.3 (5)
C14—C10—C11—N2	−0.9 (4)	C12—C13—C14—C10	−1.1 (4)
C9—C10—C11—N2	178.0 (3)	C12—C13—C14—Cl1	178.5 (2)
C4—N1—C5—C6	−0.1 (5)	C11—C10—C14—C13	1.6 (4)
C4—N1—C5—Cl3	−179.7 (2)	C9—C10—C14—C13	−177.3 (3)
C7—C6—C5—N1	0.6 (5)	C11—C10—C14—Cl1	−178.0 (2)
C7—C6—C5—Cl3	−179.8 (2)	C9—C10—C14—Cl1	3.1 (4)
C5—C6—C7—C3	−0.9 (5)	C1—O1—C2—O2	3.5 (5)
C5—C6—C7—Cl4	179.3 (2)	C1—O1—C2—C3	−176.6 (3)
C4—C3—C7—C6	0.7 (4)	C7—C3—C2—O2	11.1 (5)
C2—C3—C7—C6	−178.5 (3)	C4—C3—C2—O2	−168.0 (3)
C4—C3—C7—Cl4	−179.5 (2)	C7—C3—C2—O1	−168.8 (3)
C2—C3—C7—Cl4	1.3 (4)	C4—C3—C2—O1	12.0 (4)
C8—O4—C9—O3	1.1 (5)	C11—N2—C12—C13	0.9 (5)
C8—O4—C9—C10	−178.8 (3)	C11—N2—C12—Cl2	−178.8 (2)
C11—C10—C9—O3	−171.2 (3)	C14—C13—C12—N2	−0.2 (5)
C14—C10—C9—O3	7.7 (5)	C14—C13—C12—Cl2	179.5 (2)

### Hydrogen-bond geometry (Å, °)

**Table:**

*D*—H···*A*	*D*—H	H···*A*	*D*···*A*	*D*—H···*A*
C6—H6A···O3^i^	0.93	2.41	3.309 (4)	162
C11—H11A···O2^ii^	0.93	2.60	3.513 (4)	168

Symmetry codes: (i) −*x*+1, *y*−1/2, −*z*+3/2; (ii) *x*−1, *y*, *z*−1.
